# Pore Scale Visualization of Drainage in 3D Porous Media by Confocal Microscopy

**DOI:** 10.1038/s41598-019-48803-z

**Published:** 2019-08-26

**Authors:** Débora F. do Nascimento, José R. Vimieiro Junior, Sidnei Paciornik, Marcio S. Carvalho

**Affiliations:** 1 Department of Mechanical Engineering, Pontifícia Universidade Católica do Rio de Janeiro, Rio de Janeiro, RJ 22451-900 Brazil; 2 Department of Chemical and Materials Engineering, Pontifícia Universidade Católica do Rio de Janeiro, Rio de Janeiro, RJ 22451-900 Brazil

**Keywords:** Engineering, Fluid dynamics

## Abstract

We visualize the dynamics of immiscible displacement of a high viscosity wetting phase by a low viscosity non-wetting phase in a three-dimensional (3D) glass bead packing using confocal microscopy. Both phases were doped with two different fluorescent dyes, which enabled visualization of both phases simultaneously and quantification of the phase volumes without the need of image subtraction operations. The transient results show details of the displacement process and how pores are invaded by the non-wetting displacing phase. The static images at the end of the displacement process reveal how the trapped ganglia volume and morphology change with capillary number. The wetting phase is trapped as pendular rings spanning one to multiple pore necks. Details of the pore scale flow of oil wet media revealed with the experimental methods presented here can lead to better fundamental understanding of the physical processes and optimized enhanced oil recovery methods, CO_2_ sequestration and aquifer remediation.

## Introduction

The displacement of one fluid by another immiscible fluid in a porous media is crucial in many applications, including oil recovery^1–3^, CO_2_ sequestration^4,5^, ground water remediation^6^, impregnation of catalyst support^7^, chormatography^8^, transport through tissues^9–12^, drying and impregnation of porous membranes^13,14^. Macroscopic flow description using Darcy equation does not describe pore scale details of the fluid displacement process that may have a dramatic impact on all these processes. The pore-scale displacement pattern can be highly disordered because of the viscosity ratio between the displacing and displaced fluids and the complex structure of the pore space. The sequence of pore invasion and the trapped volume of the displaced phase depends on the viscosity ratio between the liquid phases, the ratio between viscous to capillary forces, the geometry and wettability of the porous space.

Macroscopic multiphase flow phenomena are directly associated to the complex multiphase flow at the microscopic scale. Recent developments on flow visualization techniques have led to a growth on the use of pore-scale flow visualization to gain detailed information on the different phases’ occupation of the pore space and volume and morphology of the trapped displaced ganglia. Different techniques have been used to visualize pore-scale phenomena during the displacement process, using two-dimensional transparent micromodels and optical and fluorescent microscopy^15,16^, nuclear magnetic resonance (NMR)^17,18^ and X-ray micro-CT^19–22^ in three-dimensional porous media. However, fast visualization is typically challenging, and space and time resolution in 3D experiments are usually limited. Recently, Datta and collaborators^23–28^ have applied confocal microscopy as an approach to visualize pore-scale dynamics and the intricate structure of trapped ganglia, within a 3D porous medium.

The displacement of a non-wetting phase is called imbibition and has been extensively analyzed. Using confocal microscopy to visualize the 3D phase distribution, Datta *et al*.^27^ have shown that as the viscous forces become stronger, i.e. as capillary number rises, ganglia above a certain size are mobilized and removed, and the total volume of trapped non-wetting phase falls. The ganglia configuration is set by the balance between the viscous force exerted on the ganglia and the capillary trapping force. Oughanem *et al*.^29^ used X-ray tomography to study the oil ganglia distribution in water-wet sandstone plugs and the effect of pore structure on the ganglia morphology and volume of trapped oil as a function of capillary number.

Despite being extremely relevant in oil recovery, since most oil reservoirs are not purely water-wet and display mixed-wetting conditions associated to the sorption of surface-active components in the oil to the rock surface, the displacement of the wetting phase, called drainage, is not as well understood as imbibition. The fluid displacement dynamics in drainage is remarkably different from imbibition. To displace a wetting phase from a pore, the pressure in the invading non-wetting phase has to be above a threshold capillary pressure, which is a function of the interfacial tension between the phases and the pore geometry. The wall wettability has a dramatic impact on the efficiency of the displacement process in oil recovery processes. Avendaño *et al*.^30^ compared the sequence of events that occur in oil displacement processes in water-wet and oil-wet 2D porous media micromodel to show that, at low capillary number, the remaining oil volume was larger in water wet media. The results agree with the core flooding experiments of Jadhunandan and Morrow^31^ and the capillary network model of Zhao *et al*.^32^. Iglauer *et al*.^1^ studied the size distribution of trapped oil ganglia in oil- and water-wet sandstone using X-ray tomography. They showed that the oil ganglia had a flatter, sheet-like configuration in the oil-wet media while they were more spherical in the water-wet case. In the oil-wet sandstone, the trapped oil ganglia were mostly located adjacent to the walls and in smaller pores, leading to a smaller volume of trapped oil. Details of the dynamics of the liquid displacement and morphology of the trapped ganglia are still not clear.

Here, we use fast confocal microscopy to visualize the dynamics of the fluid displacement flow during drainage. We doped both the wetting, displaced phase and the non-wetting, invading phase, with different fluorescent dyes, which enabled the visualization of both phases simultaneously inside the pore space. The ganglia size distribution and detailed information about morphology of the trapped wetting phase within a three-dimensional porous media is quantified as a function of flow conditions.

## Results and Discussion

**Figure 1:**
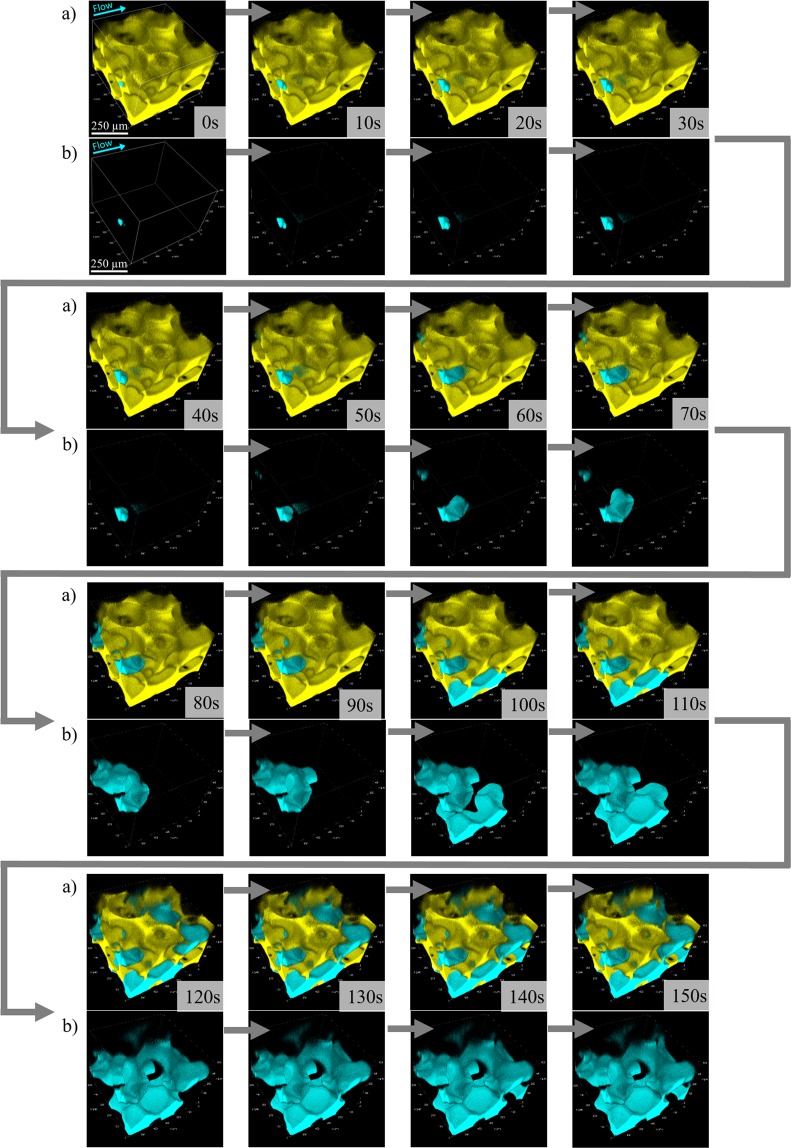
3D rendering images showing the dynamic behavior of drainage. (**a**) Showing both the displaced wetting (yellow) and displacing non-wetting (blue) phases and (**b**) showing only the non-wetting liquid.

Figure 1 shows the 3D rendering of the displacement process dynamics in an 0.581 × 0.581 × 0.400 mm^3^ volume of the porous media. As mentioned before, because the fluorescent dyes used in both phases emit at different wave lengths, it was possible to visualize both phases simultaneously. The wetting displaced phase is shown in yellow and the non-wetting invading liquid is shown in blue in Fig. 1 and in the Supplementary Videos 1 and 2. The sequential images were acquired every 10 seconds. Figure 1a,b show, respectively, both phases together and only the non-wetting fluid, in blue. The flow rate imposed at this particular case was *Q* = 0.027 mL/h, which corresponded to a capillary number of *Ca* = 1 × 10^−6^.

**Figure 2:**
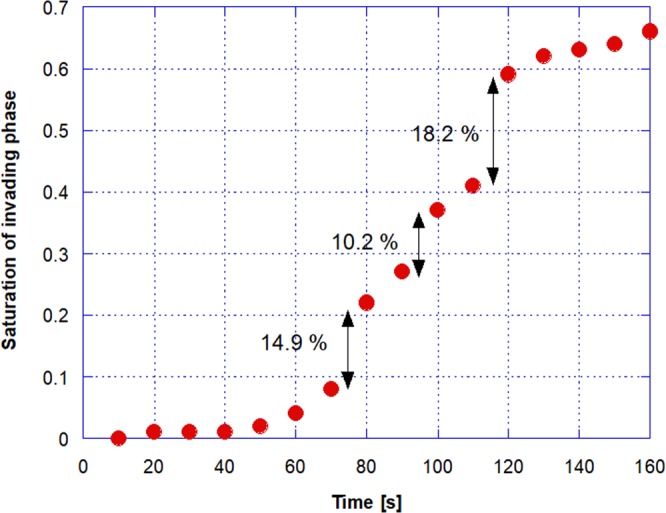
Evolution of the non-wetting invading phase saturation showing Haines jump.

The images reveal details of the pore-scale physical mechanisms involved in drainage and enables quantitative analysis of the invasion rate, temporal evolution of saturation and morphology of the trapped ganglia. These parameters are important for evaluating the effectiveness of different types of tertiary recovery processes^33^. As expected, the saturation changes occur in burst (Haines jump)^34–36^, whenever the pressure difference between the phases surpasses the capillary pressure. Figure 2 presents the evolution of the displacing liquid saturation. The Haines jump are clear at *t* = 70, 90 and 110 *s*, with large saturation changes at these times.

The final wetting phase (yellow) saturation is close to *S*_*wr*_ = 33.8%. This relative high value was expected because of the low capillary number. The viscous forces were not high enough to overcome capillary forces and mobilize a larger amount of the displaced phase^37^.

**Figure 3:**
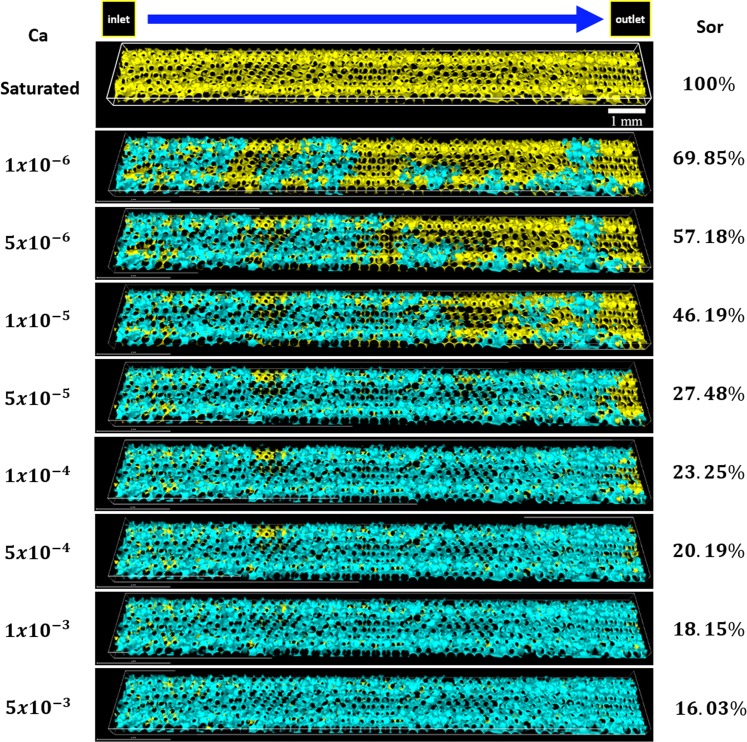
Rendering of  3D images acquired at steady state after the injection of the displacing fluid at different capillary numbers, which contemplates both phases: wetting (yellow) and non-wetting (blue) fluids.

**Figure 4:**
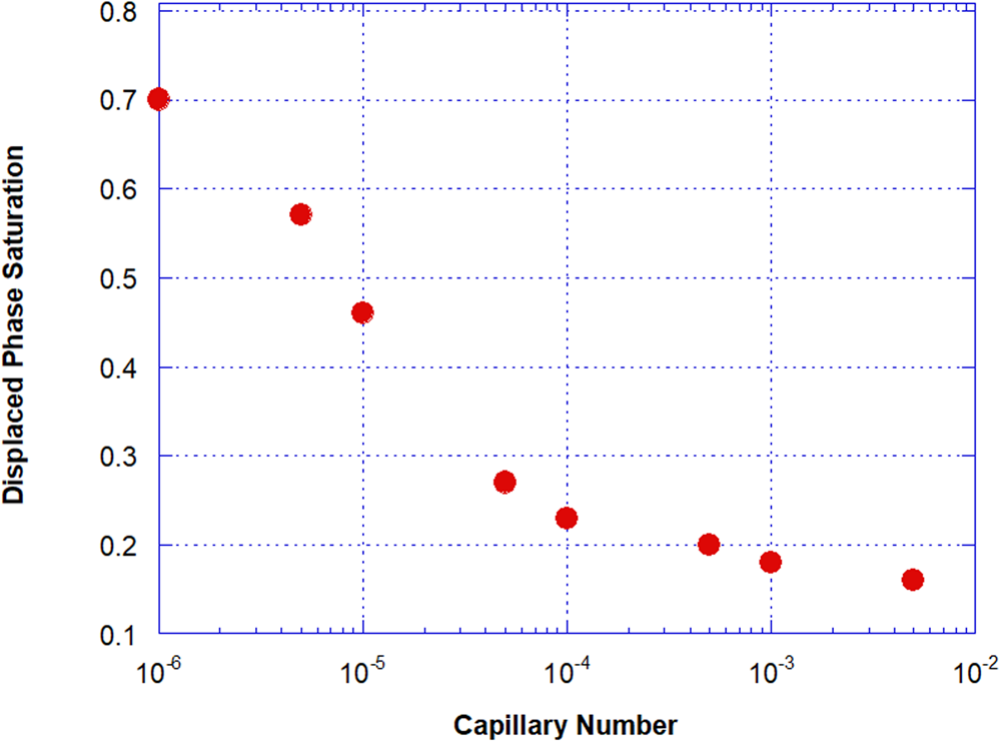
Saturation of the displaced phase as a function of the capillary number.

The resulting steady-state phase distribution over a large volume of the porous medium after the injection of the displacing liquid at different capillary numbers is presented in Fig. 3. Both the wetting (yellow) and non-wetting (blue) phases are shown. The trapped volume of the displaced phase was determined by image processing. The remaining displaced phase saturation *S*_*wr*_ as a function of capillary number is presented in Fig. 4. As expected, the volume of trapped ganglia falls as capillary number rises. It falls from *S*_*wr*_ ≈ 0.7 to *S*_*wr*_ ≈ 0.16 as capillary number rises from *Ca* = 1 × 10^−6^ to *Ca* = 5 × 10^−3^. Trapped displaced phase ganglia is mobilized if the viscous forces acting on them exceed the capillary retaining forces^38^. The remaining saturation of the displaced fluid is still relatively high at the largest capillary number explored. This observation agrees with data from porous media formed with smooth spherical beads, which reveals that the residual wetting phase saturation could not be reduced below *S*_*wr*_ ≈ 0.09^39^.

**Figure 5:**
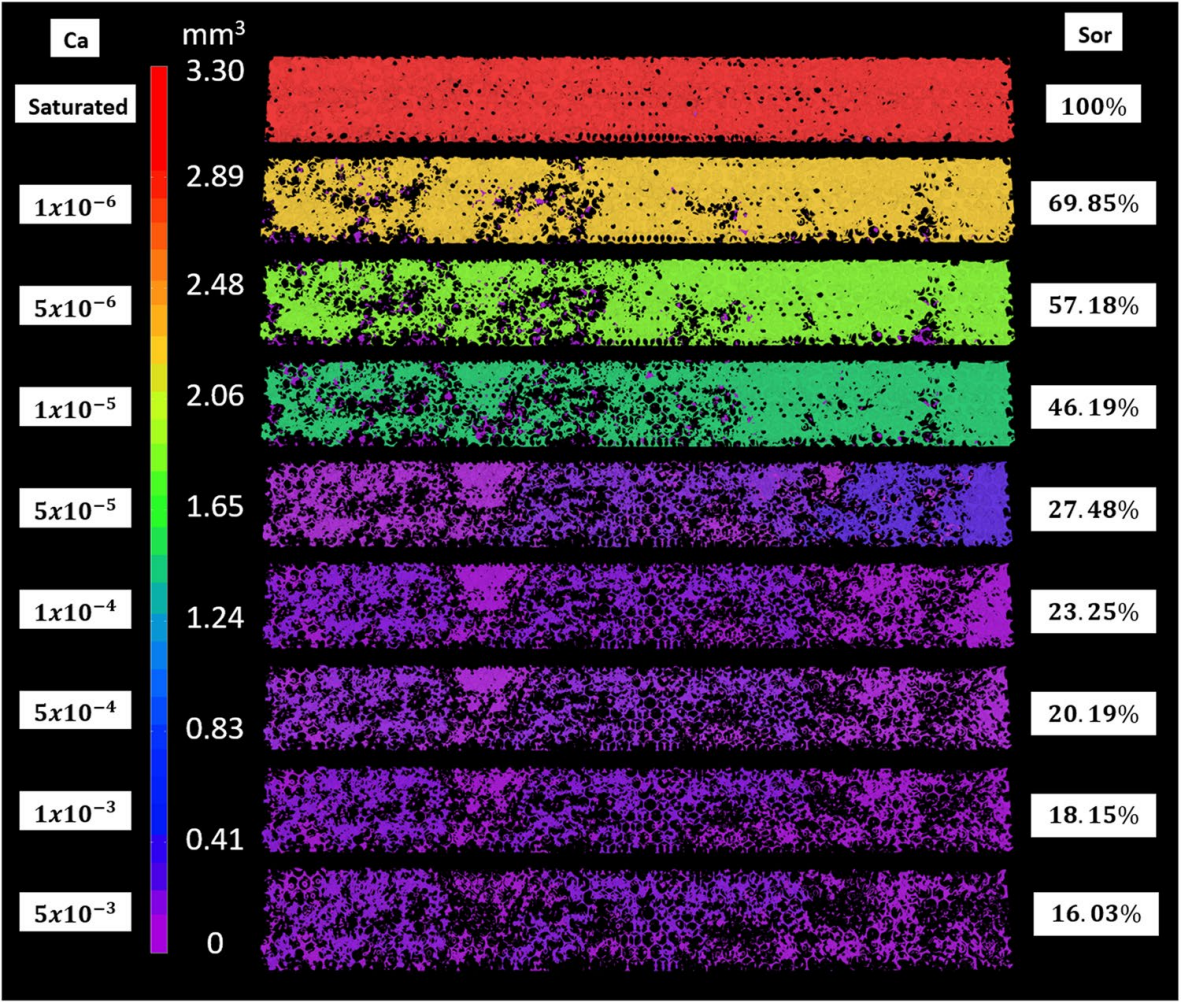
Volume of trapped displaced phase ganglia as a function of capillary number.

**Figure 6:**
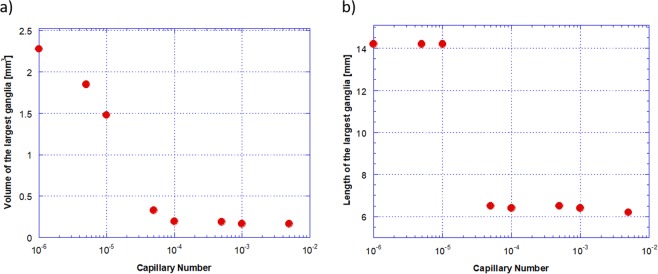
Geometric parameters of the largest trapped displaced phase ganglia as a function of capillary number (**a**) volume and (**b**) length along the flow direction.

3D high resolution images enable quantitative analysis of the intricate morphologies of the trapped displaced phase ganglia at each flow condition. Figure 5 presents the images of the trapped displaced phase ganglia color-coded based on their volume. At *Ca* > 1 × 10^−5^, only small ganglia remain trapped. The capillary number is large enough to mobilize any large ganglia. The volume of the largest ganglia at each flow condition, shown in Fig. 6a, decreases with capillary number. At *Ca* ≈ 1 × 10^−5^ there is a large decrease on the volume; above *Ca* ≈ 1 × 10^−4^, the volume of the largest ganglia remains almost constant. The length along the flow direction of the largest ganglia as a function of capillary number is shown in Fig. 6b. For *Ca* < 1 × 10^−5^, the largest ganglia spans the entire imaged region, *L* ≈ 14 *mm*. A large reduction is observed at *Ca* ≈ 5 × 10^−5^. Above this value, the largest length remains almost constant and equal *L* ≈ 6 *mm*.

**Figure 7:**
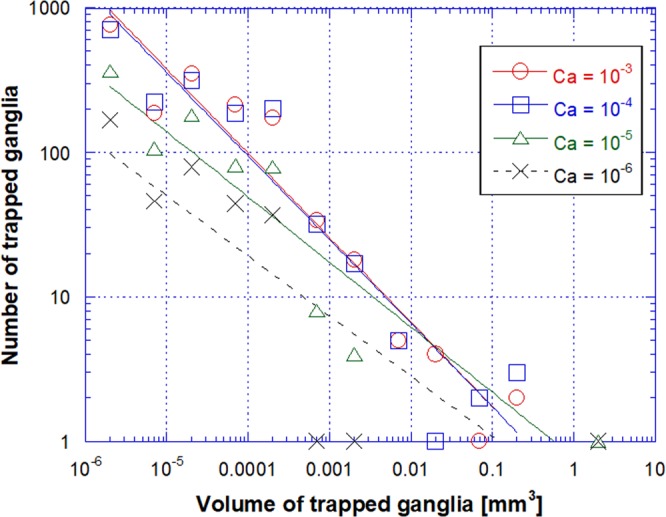
Trapped ganglia size distribution as a function of capillary number.

At each value of capillary number, there is a broad distribution of trapped ganglia volume. We defined two classes of ganglia volume per decade and Fig. 7 presents the number of ganglia in each volume class for different capillary numbers. For *Ca* < 1 × 10^−5^, there is 1 large ganglia with a volume larger than 1 mm^3^. For all flow rates, the number of trapped ganglia present at each size class falls with the volume; there is a large number of small ganglia and a small number of large ganglia. The behavior is well described by a power-law relation: *N* ≈ *V*^*−n*^, with *n* ≈ 0.44 at *Ca* < 1 × 10^−5^, and *n* ≈ 0.58 at *Ca* > 1 × 10^−4^. Power-law behavior was also observed in experiments in sandstones^28,32^ using X-Ray tomography images and 2-D micromodels^28^, with different values of the exponents. Iglauer *et al*.^1^ found *n* ≈ 2.12 in oil-wet sandstones and Avendaño *et al*.^30^ reported *n* ≈ 0.65 in oil-wet 2D glass micromodel. One possible reason for the different exponents is the very different pore structures of the porous media in these analyses. The pore size distribution in a sandstone is much broader than in the 2D micromodel and in the monodispersed glass bead packing.

**Figure 8:**
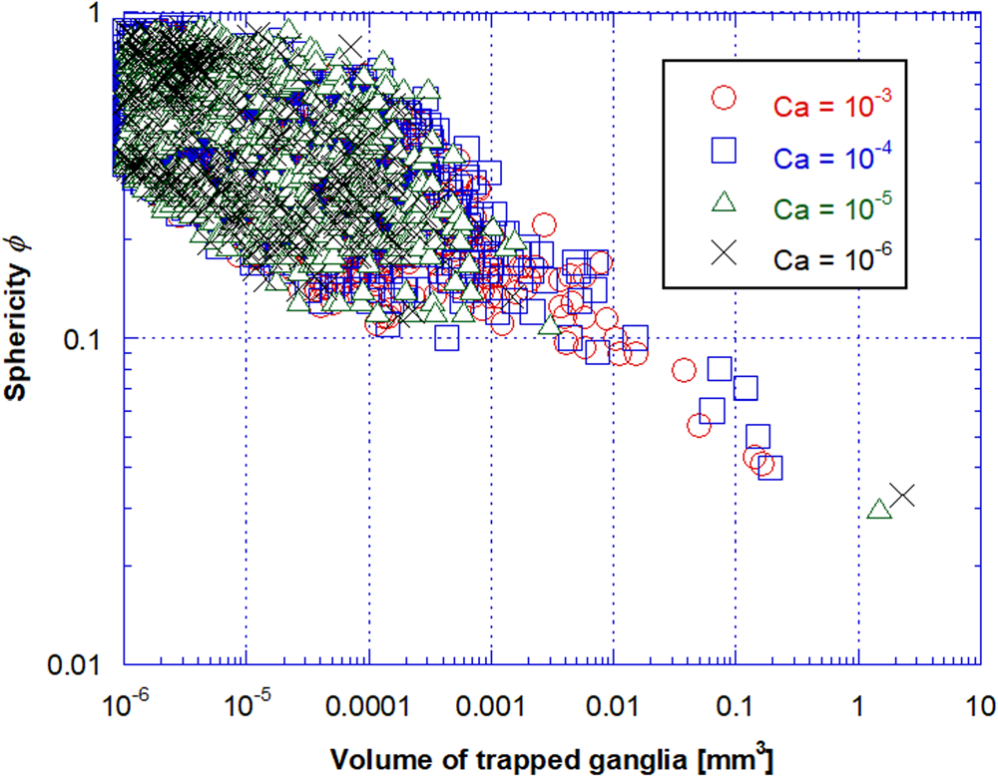
Sphericity of trapped ganglia as a function of their volume for different capillary numbers.

In order to study the shape of the trapped ganglia, we plot in Fig. 8 the sphericity *φ* of each individual ganglion as a function of its volume for different capillary numbers. Sphericity, defined as $\varphi ={(6{\pi }^{\tfrac{1}{2}}{V}_{p})}^{\tfrac{2}{3}}/{A}_{p}$ (*V*_*p*_ and *A*_*p*_ are the volume and surface area of the ganglion), measures how closely the shape of an object approaches a sphere. By definition, the sphericity of a sphere is *ϕ* = 1 In general, the sphericity of a trapped ganglion falls as its volume becomes larger, indicating that the large ganglia have a relatively high surface area, with *ϕ* < 0.1 for ganglia with volume lower than *V*_*p*_ ≈ 0.01 *mm*^3^, and with a shape far from spherical. This result agrees with Iglauer *et al*.’s^20^ finding. They reported that the oil ganglia in an oil-wet sandstone had a flatter, sheet-like configuration while they were more spherical in the water-wet case. The dispersion of the sphericity value increases as the ganglia volume falls. The shape of the very small ganglia approaches that of a sphere with values of sphericity close to 1.

**Figure 9:**
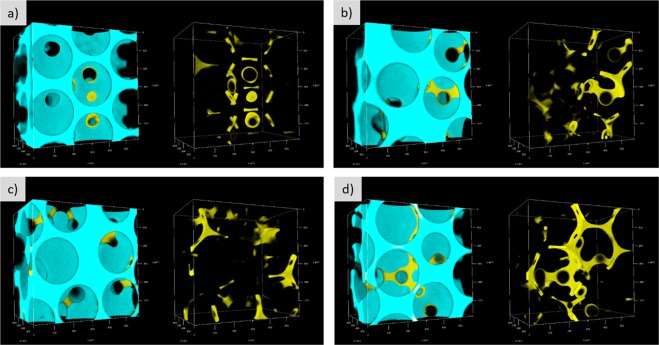
3D images showing both the displacing non-wetting (blue) and displaced wetting (yellow) phases in four different positions along the porous media to exemplify different ganglia morphology. The trapped ganglia are in the form of pendular rings, spanning one, three and multiple pore necks.

Since the wetting phase preferentially wets the glass beads, the majority of the trapped fluid formed pendular rings^40^, as shown in Fig. 9. These are small volumes of fluid forming axisymmetric liquid bridges with interfaces (meniscus) between two or more axisymmetric solids^41^. These individually isolated segments of fluid surrounding the bead-to-bead contact points are shown in Fig. 9. The images were obtained at *Ca* = 5 × 10^−3^ and represent different positions in the porous media after drainage is completed. They show examples of structures spanning one, three and multiple pore-necks. The high-resolution 3D confocal images allowed visualization of these pendular structures.

**Figure 10:**
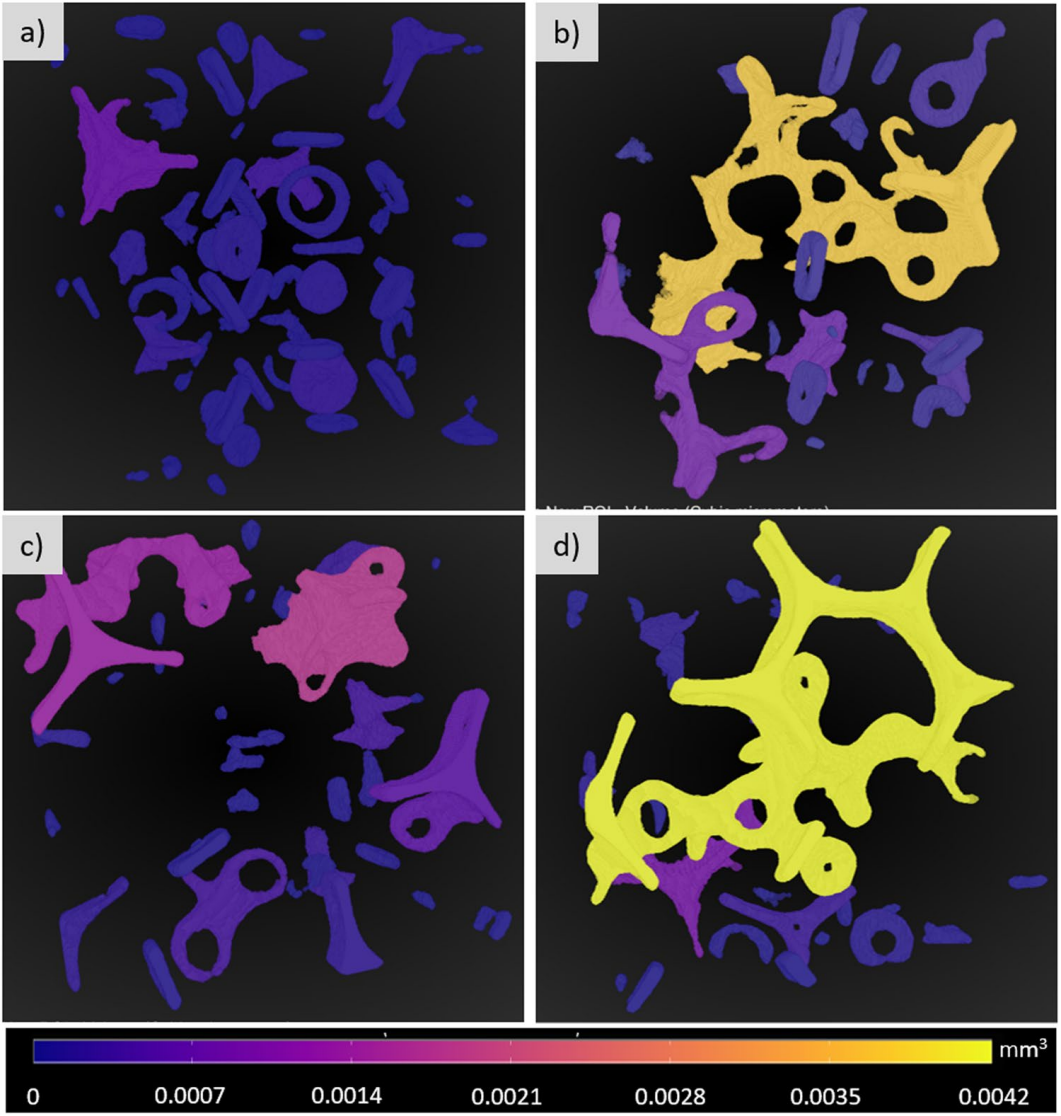
3D processed images with ganglia volume colored according to the scale bar.

Pendular rings were analyzed previously only by numerical simulation, and experimentally by X-ray computed micro-tomography and synchrotron^40,42–47^. Figure 10 shows the same images of Fig. 9 with the trapped wetting phase color-coded by their volume. The volume is proportional to the number of pore necks that the ganglion spans. The orange and yellow structures (large volumes) of Fig. 10 span more than 4 bead-to-bead contacts.

### Final remarks

This works presents visualization using confocal microscopy of the 3D pore scale dynamics during drainage in a glass bead packing. The two phases were doped with different fluorescent dyes, which enabled the visualization of both phases simultaneously. The transient results show Haines jumps associated with the displacement of the wetting by the non-wetting phase. At the end of the displacement process at different capillary numbers, the trapped wetting phase ganglia was analyzed to determine the ganglia size distribution, its morphology and the total trapped volume.

The volume of the trapped displaced phase falls as capillary number rises. One of the reasons is that the volume of the largest ganglia falls considerably above a critical capillary number. The ganglia size distribution follows a power-law relation, as observed by X-Ray tomography in sandstones. The images reveal that the trapped wetting phase form pendular structures spanning a single or multiple pore necks.

Fundamental understanding of the dynamics of the pore scale drainage flow brings new insights on how to reduce the residual oil saturation on oil wet reservoirs and consequently improve oil recovery.

## Materials and Methods

**Figure 11:**
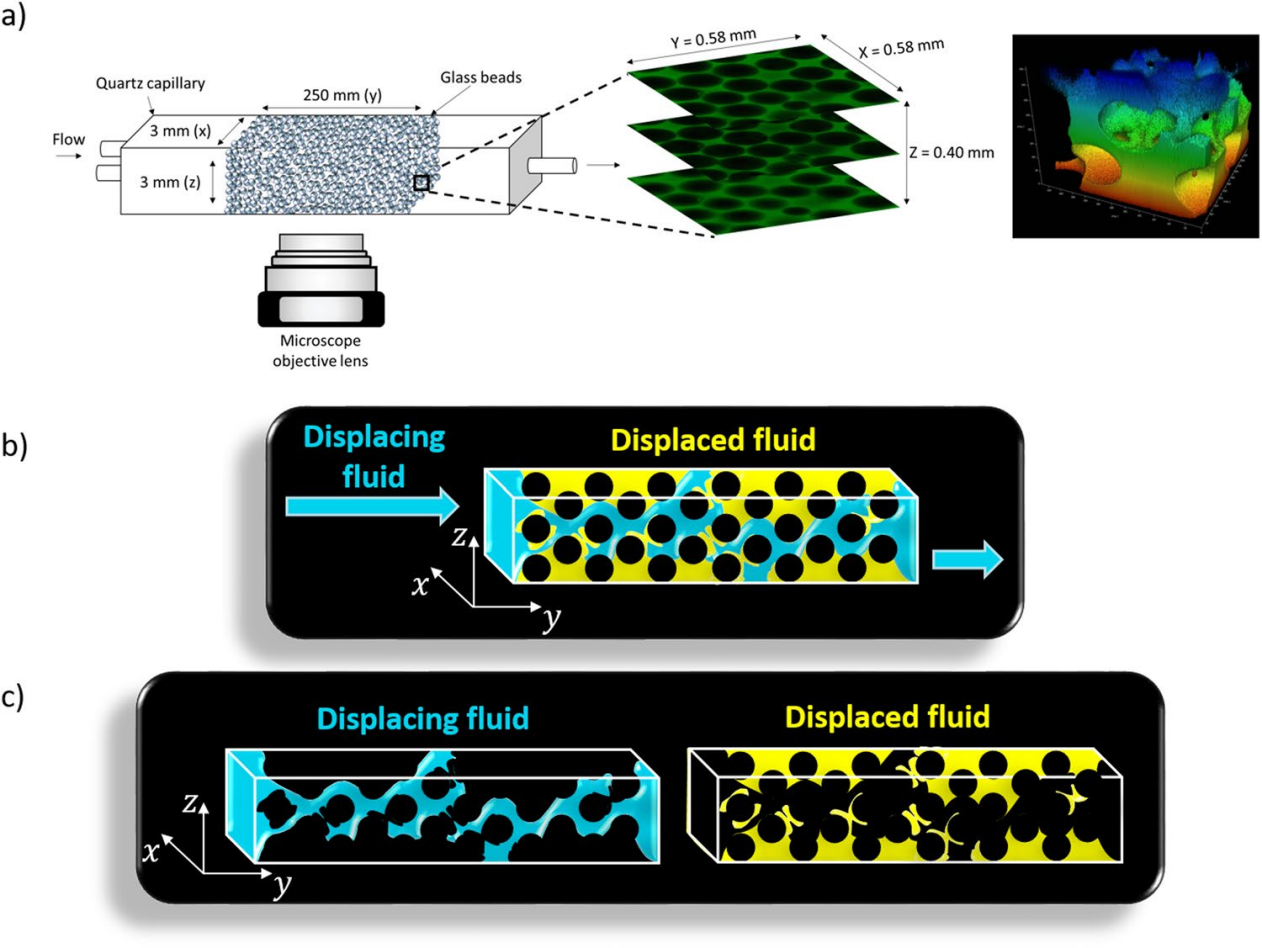
Schematic illustrations of (**a**) image acquisition and 3D rendering process; (**b**) the wetting fluid (yellow) been displaced by the non-wetting fluid (blue); and (**c**) residual fluids after the drainage process.

The 3D transparent porous media was prepared by sintering packed hydrophilic glass beads, with radii of *r* = 128 ± 2.8 µm (Mo-Sci, polydispersity less than 2.2%), in thin walled squared quartz capillary (Friedrich & Dimmock, Inc.), with cross sectional area of 9 mm² and length of 25 mm. The total volume of the porous medium was 225 mm^3^. To visualize the 3D pore structure and the drainage dynamics, an SP8 confocal microscope from Leica Microsystems was used. Image acquisition was restricted to an area away from each edge of the medium to minimize boundary effects. We used two modes of visualization. The first was a static mode, which revealed the final phase distribution at the end of the displacement process. The 3D structure with approximately 400 μm in depth (z-direction) were constructed from 168 optical slices in the x-y plane, spaced by 2.407 µm from each other along the z-direction, as sketched in Fig. 11a. Each stack spanned an area of 27.45 mm^2^, which corresponded to 36.6% of the total lateral area of the porous media, and was composed by 27 × 3 images with 512 × 512 pixels with a resolution of 1.137 × 1.137 µm. The total volume of the imaged region was of 11.1 mm^3^, which corresponded to 4.93% of the total volume. The acquisition time of each stack was approximately 76 seconds and of the entire imaged volume was 103 minutes. The second visualization mode was used to study the dynamics of the displacement process. Each 3D image with approximately 400 μm in depth (z-direction) were constructed from 40 optical slices in the x-y plane spaced by 10 µm along z-direction. Each stack consisted of one image with 512 × 512 pixels with a resolution of 1.137 × 1.137 µm. The acquisition time for the entire 3D image was approximately 10 seconds. The fast acquisition time enabled the visualization of dynamic events with a speed lower than approximately 60 μm/s. This speed is much higher than usual displacement velocity in oil recovery processes, which is approximately 3 μm/s (30 cm/day).

To avoid the scattering of light from the surface of the glass beads, we formulated the injected (non-wetting) and displaced (wetting) phases to match their refractive index with that of the glass beads (*n* = 1.478). The phases compositions were based on the fluids used by Krummel *et*. *al*.^23^. Other components were added in order to not only match the refractive index of the glass beads, but also to assure compatibility with both fluorescent dyes and to obtain a suitable viscosity ratio between the phases, with the displaced phase being more viscous than the injected phase. In this way, our experiments represent an idealized model of oil displacement by water injection in an oil-wet porous media.

The non-wetting liquid was a mixture of 84.59 wt.% of an immersion optical oil (code 5040, Cargille Labs) and 15.41 wt.% of a commercial lubricant oil (Waynoil, Schulz). A fluorescent dye, Oil-Glo 22^®^ (Spectroline), was added to allow the visualization with confocal microscopy. The viscosity of the non-wetting, injecting phase was *μ*_*nw*_ = 20.08 mPa.s at 24 °C. The wetting liquid was a mixture of 31.87 wt.% of sucrose, 54.01 wt.% of dimethyl sulfoxide, 13.72 wt.% of Milli-Q^®^ water and 0.39 wt.% of fluorescent dye Methylene Blue (Sigma Aldrich). The viscosity of the wetting, displaced phase was *μ*_*nw*_ = 33.68 mPa.s at 24 °C. The viscosities were evaluated with a stress-controlled rheometer, at a constant shear rate of 10 s^−1^. The viscosity ratio between the displaced and injected liquids was *μ*_*w*_/*μ*_*nw*_ = 1.68. The interfacial tension was σ = 17 mN/m, measured using du Noüy ring. The contact angle between the wetting phase and the glass beads was approximately 65°.

The experimental procedure was based on previous analysis using confocal microscopy^23–27^. Before each experimental run, the porous medium was initially saturated with CO_2_ gas during 2 hours at 10 psi. Then, the wetting fluid was injected using an 11 Elite Plus Syringe pump (Harvard Apparatus) and Gastight glass syringes (Hamilton) to fully saturate the porous medium.

The effective porosity, measured by the weight difference of the fully saturated and dry medium, was of *ϕ* = 0.421 ± 0.001. The effective porosity obtained by evaluating the volume of the wetting phase using the confocal 3D image was *ϕ* = 0.407 ± 0.007. The absolute permeability was *K* = 17.87 D, which was determined by Darcy’s law, using the values of the pressure drop across the porous medium as a function of the flow rate, measured with a DPGW-07^®^ digital pressure gauge (Dwyer).

The non-wetting, displacing phase (blue in Fig. 11b,c) was injected at different flow rates, varying from *Q*_*inj*_ = 0.027 to *Q*_*inj*_ = 137.15 mL/h, leading to a capillary number range of *Ca* ≡ *μ*_*nw*_*Q*_*inj*_/*Aσ* = 10^−6^ to 5 × 10^−3^. At each condition, the dynamics of drainage was observed and the phase distribution at the end of the injection process was measured, as sketched in Fig. 11b,c. Since both phases were fluorescent at different wavelengths, we could visualize the distribution of each phase without the need of the image subtraction operation used in previous works^23–25,27^, which compromises the accuracy of the analysis.

The 3D images were processed to extract numerical attributes and quantitative analysis using the software FIJI/ImageJ^®^, which is a Java-based image processing program developed at the National Institutes of Health (NIH) and the Laboratory for Optical and Computational Instrumentation (LOCI, University of Wisconsin)^48,49^, and Dragonfly^®^ (ORS)^50^.

**Figure 12:**
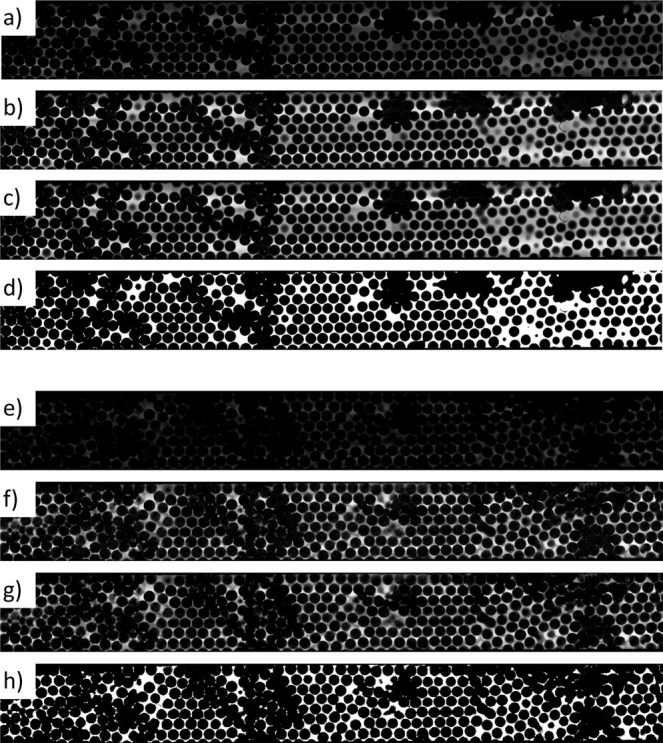
Image processing sequence for two representative slices. Top 4: Slice 1, at the bottom wall. Bottom 4: Slice 80, 192 µm depth. (**a**,**e**) Original images. (**b**,**f**) After histogram equalization. (**c**,**g**) After noise reduction with Sigma filter. (**d**,**h**) After segmentation with the Otsu method.

A FIJI macro was prepared to process all images in the volume. The sequence of operations is illustrated in Fig. 12 for two representative slices, one at the bottom wall and another at 192 µm depth. The latter shows much lower contrast. The first step involved histogram equalization^51^ to make different image layers have similar brightness and contrast. This was critical for the segmentation step, as described in the following. Then, noise was reduced with the so-called Sigma filter^52^, which is an edge preserving low-pass filter. Finally, the fluids were automatically discriminated, layer by layer, using the segmentation Otsu´s method. Here, the similarity of the intensity histograms achieved by the equalization step was critical to allow accurate segmentation in all layers without the need for manual adjustments.

## Supplementary information

Video1

Video 2
