# Supplementary figures and images for: Pore Scale Visualization of Drainage in 3D Porous Media by Confocal Microscopy
Source: Sci Rep. 2019 Aug 26;9:12333. doi: 10.1038/s41598-019-48803-z (PMC6710249; doi:10.1038/s41598-019-48803-z)

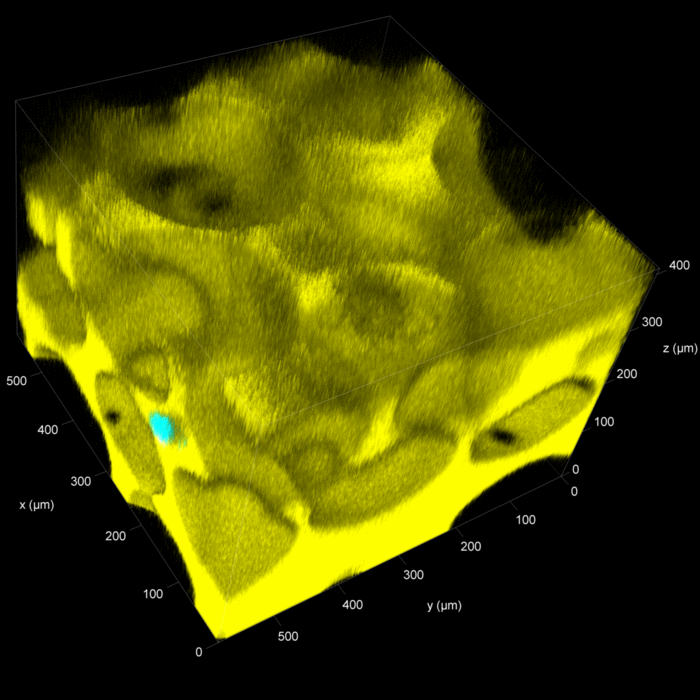

Supplement: Supplementary file 1 — Video1 [file 41598_2019_48803_MOESM1_ESM.gif]

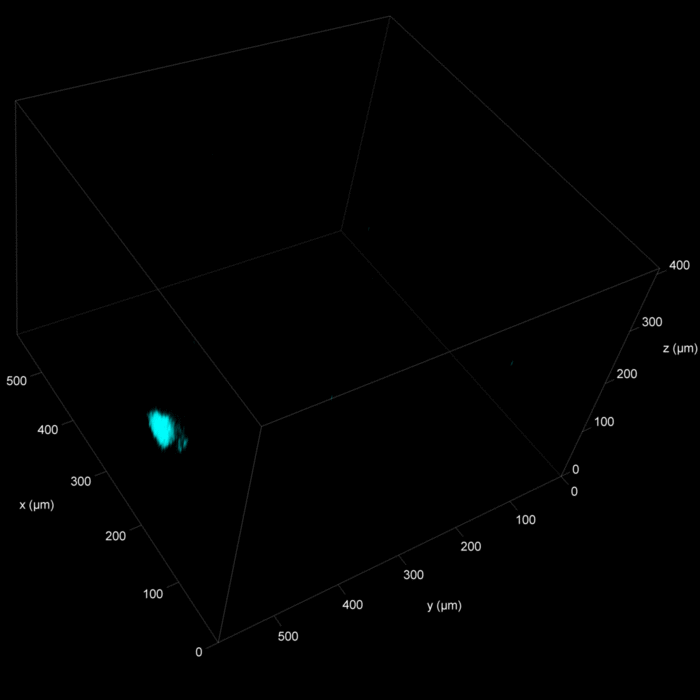

Supplement: Supplementary file 2 — Video 2 [file 41598_2019_48803_MOESM2_ESM.gif]
